# Everybody's Page

**Published:** 1893-01-28

**Authors:** 


### Everybody's Page.

The task of biographer to Sir Richard Owen has fallen on his son, the Rev. R. Owen, the publication of which work will doubtless be eagerly looked for.

Some model dwelling-houses are in the course of erection at Vienna for the use of poor workmen. These houses are to be constructed on the latest and most approved sanitary methods, ventilation being given much attention to. The workmen, it is agreed, will pay 4 per cent. on the buildings, and within a period of twenty-five years the lodger will become the permanent owner.

Lord Salisbury has undertaken to preside at a large public meeting at Oxford on March 1st, in aid of the funds required for the extension and improvement of the Radcliffe Infirmary. The system of presenting purses containing specified sums will be put into operation in this case; let us hope with the same success as it met with at the recent gathering at St. Mary's Hospital, Paddington.

A contributor to the pages of the *San Francisco Monitor* has displayed some originality in his treatment of "Foreign Titles and American Girls." The Anglo-American Alliance of cheque-books and titled debts has, up to now, been accepted as a fair and two-sided bargain (if one may use the expression), wherein both parties have equally open eyes, but this is not the real case, asserts the writer in the *San Francisco Monitor.* He draws a picture from the other side of the Atlantic suggestive to our minds of an unceasing arrival at American shores of large cargoes of evil, bankrupt foreigners, who have come to carry off, against their will, these young and unsophisticated millionairesses, and to the most certain wreckage of their future happiness. But is not the remedy in their own hands? The door which America has, with such lavish hospitality, thrown open to the scions of European aristocracy can surely in the future be bolted and barred from within.

It is with a mixed sense of amusement and relief that we read of a new and rebellious League which John Strange Winter is leading against Crinoline. For a quarter of a century now we have been pleased to look back on the days of "hoops" with a kindly toleration born of pity. "Poor things, they knew no better," we would content ourselves by reflecting, as we gazed at our grandmother's voluminous *entourage* in a faded photograph. "Such encumbrances were in the pre-historic times of female athleticism, of female enlightenment generally." In some such manner have we ever been ready to smooth down our ruffled sensibilities concerning the modes of past ages. But what have we to say for women, who, claiming equal rights of civilisation with man, can even entertain the idea in this year of grace 1893, of outraging the first principles of progress by marching in a retrograde manner. Or is it, as John Strange Winter has it, in the letter she has written concerning this coming evil, that "these things are ordered by a small clique of men; this is an established fact." Surely, then, women would do well to remember that the leaders are not the sufferers in this case, and that if men are, as we hear, the theorisers, the burden of their decrees falls not on their shoulders, but on those of the opposite sex. John Strange Winter has done her best to help womankind. She has shown a shrewd knowledge of the ways of those with whom she is dealing when, in the event of their own personal strength failing, she has suggested a post-card allegiance as a guarantee of abstinence from the dire evil which is foreshadowing woman's immediate future.

The cold weather at St. Petersburg, which has been almost phenomenal this season, has done some good, for it has stamped out almost all traces of the ravaging cholera epidemic.

That there is immense recuperative power in the aged after surgical operations has been shown by Dr. Blum in a recent paper in the "Archives Générales de Medecine," wherein the learned doctor gives fourteen cases of major operations performed successfully on old persons, which illustrate his opinion in a significant manner.

On Tuesday, the 31st inst., an exhibition and sale of articles, made under a cost of 6½d. each, will be held at the Morley Halls, Regent Street, W., in aid of the Ladies' Association Building Fund of the Great Northern Hospital, Holloway, N. The articles have been sent for competition, and prizes will be awarded of 10s. and 5s., and two of 2s. 6d., for young people under sixteen years of age, for the most original and useful article, or for the greatest decorative art displayed in the exhibit. Miss Amy Hill, 5, Hyde Park Mansions, N.W., is the originator of the exhibition, and will receive any articles sent for competition before the above date.

That a "little knowledge is a dangerous thing" we are taught from our infancy, though the exponents of the "popular education" of to-day are somewhat reversing this old-time adage. And with this new reversal most of the thinking world of 1893 are in sympathy. We consider that even this "little" is better than the "none" of heretofore—that in most cases it is the beginning only—and gives the taste for more. Popular education has certainly made of us more intelligent members of society, and it has opened our minds to that sense of curiosity about things around which is the commencement of all intellectual life.

A correspondent in the *Liverpool Courier* writes that the Committee of Management have not done well in their choice of date for the Hospital Sunday collection. When we consider the high figure that the united contributions of that city reached, it is not a deduction that one would make, upon first thought, but this writer bases his objections on two common sense and practical reasons; he says in the first case that the time of year chosen (the second Sunday in the New Year), is at a season when the inclemency of the weather is not propitious for crowded congregations at the seats of appeal, and further that Christmas bills have a great, besides a prior claim on our purses, and that our generosity must come second to our honesty. It will be interesting to see what the Liverpool Committee of Management will return to this.

A writer in the *Japan Mail* paints us a very distressing picture of the tortures which the female children of the better classes in China undergo for the compression of their feet. The fault of this revolting custom seems to lie at the door of the male population of China, whose ardent and professed admiration of this contortion of nature has given to the small foot a market value of its own. The actual method of this contorting is described as necessitating a long process by means of straps and ligatures for the wrenching of the sinews and the crushing of the bones of the sufferer, until the desired effect of dwarfing growth is attained. Is it not possible that the Chinese men can be civilized into considering the human foot, as nature designed it, of sufficient beauty without the operation of human interference? for in this, as in all other things, so long as there is a demand so long will there be a supply.

